# Allergic Rhinitis and Depression: Profile and Proposal

**DOI:** 10.3389/fpsyt.2021.820497

**Published:** 2022-01-04

**Authors:** Ya-Kui Mou, Han-Rui Wang, Wen-Bin Zhang, Yu Zhang, Chao Ren, Xi-Cheng Song

**Affiliations:** ^1^Department of Otorhinolaryngology Head and Neck Surgery, Yantai Yuhuangding Hospital, Qingdao University, Yantai, China; ^2^Shandong Provincial Clinical Research Center for Otorhinolaryngologic Diseases, Yantai, China; ^3^Shandong Provincial Innovation and Practice Base for Postdoctors, Yantai Yuhuangding Hospital, Yantai, China

**Keywords:** depression, allergic rhinitis, immunity, inflammation, brain

## Abstract

In addition to nasal symptoms, patients with allergic rhinitis (AR) often experience mental and psychological disorders such as depression. Depression not only makes the treatment of AR more difficult and expensive but also poses a serious impact on the patients' daily activities and quality of life, thus bringing additional burden to the families and the society. Here we systematically review the recent research advances in the correlation between AR and depression, analyze the possible causes and mechanisms of depression in AR, summarize the current diagnosis and treatment strategies, and provide our insights into the AR-related depression; in addition, we introduce briefly the basic research status on AR-related depression. We hope that this review article will provide evidence for future studies.

## Introduction

Allergic rhinitis (AR), a common chronic inflammatory disease of the upper respiratory tract, is associated with immunoglobulin E (IgE)-mediated immune-inflammatory response (1), involving multiple cells and factors, with paroxysmal sneezing, runny nose, and nasal congestion as the main clinical symptoms. It affects 10–40% of the global population (2), and its prevalence continues to increase worldwide. Although AR itself is not a life-threatening disease, it can significantly affect the quality of life and even lead to reduced work efficiency; in a certain number of patients, it is liable to trigger depression, anxiety, and other mental disorders due to the stress incurred by AR itself or other negative events, which to some extent increases the burden on families and society. It is estimated that 20.6–38.7% of AR patients will have depressive symptoms (3, 4); the treatments become more difficult and costly in AR patients with depression (5). Theoretically AR may interact with depression. While little evidence has demonstrated that depression may cause AR, an increasing number of studies have confirmed that AR will trigger depression. It has been found that the risk of AR patients developing depressive symptoms is 1.82 times that of normal individuals (6). Therefore, in addition to the treatment of the AR symptoms, early detection and management of mental problems such as depression is of great importance. However, most clinicians, even the specialists in rhinology and allergology, have limited knowledge and experience in managing depressive symptoms in AR patients, and the reasons are multifactorial but mostly due to the lack of thorough understanding of depression in AR. Here we summarize the recent research advances in depression in AR, especially the possible mechanisms and potentially effective interventions, in an attempt to provide evidence for future studies on AR-related depression.

## The Role and Classification of Depression in AR

**Figure 1:**
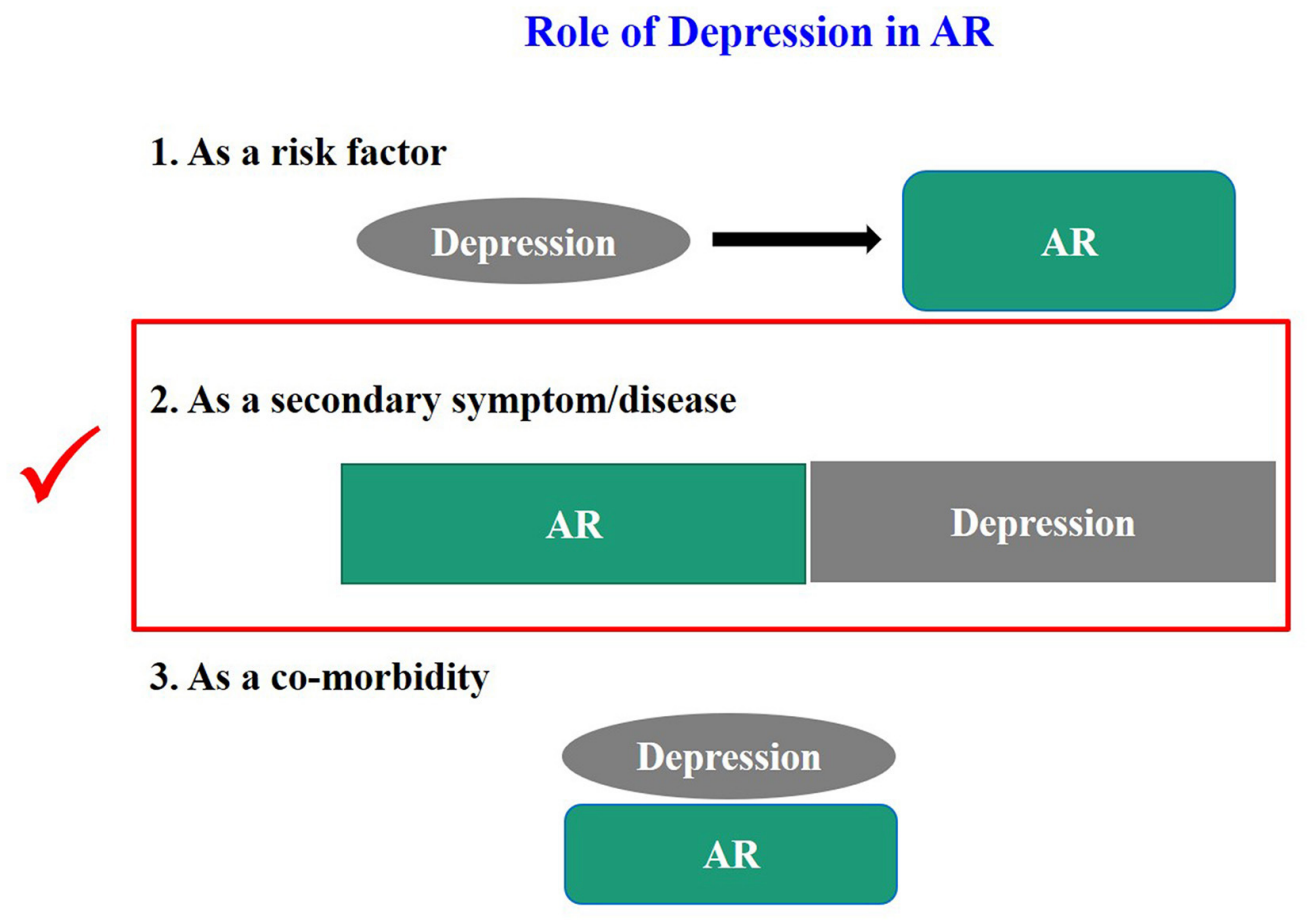
Schematic diagram of the relationship between depression and AR.

An increasing number of clinical and epidemiological investigations have found a close correlation between depression and AR (7); however, there is no definitive conclusion on such a potential link. Thus, the role of depression in AR needs to be defined. We summarize it based on the previous literature and our own experience in Figure 1.

**Figure 2:**
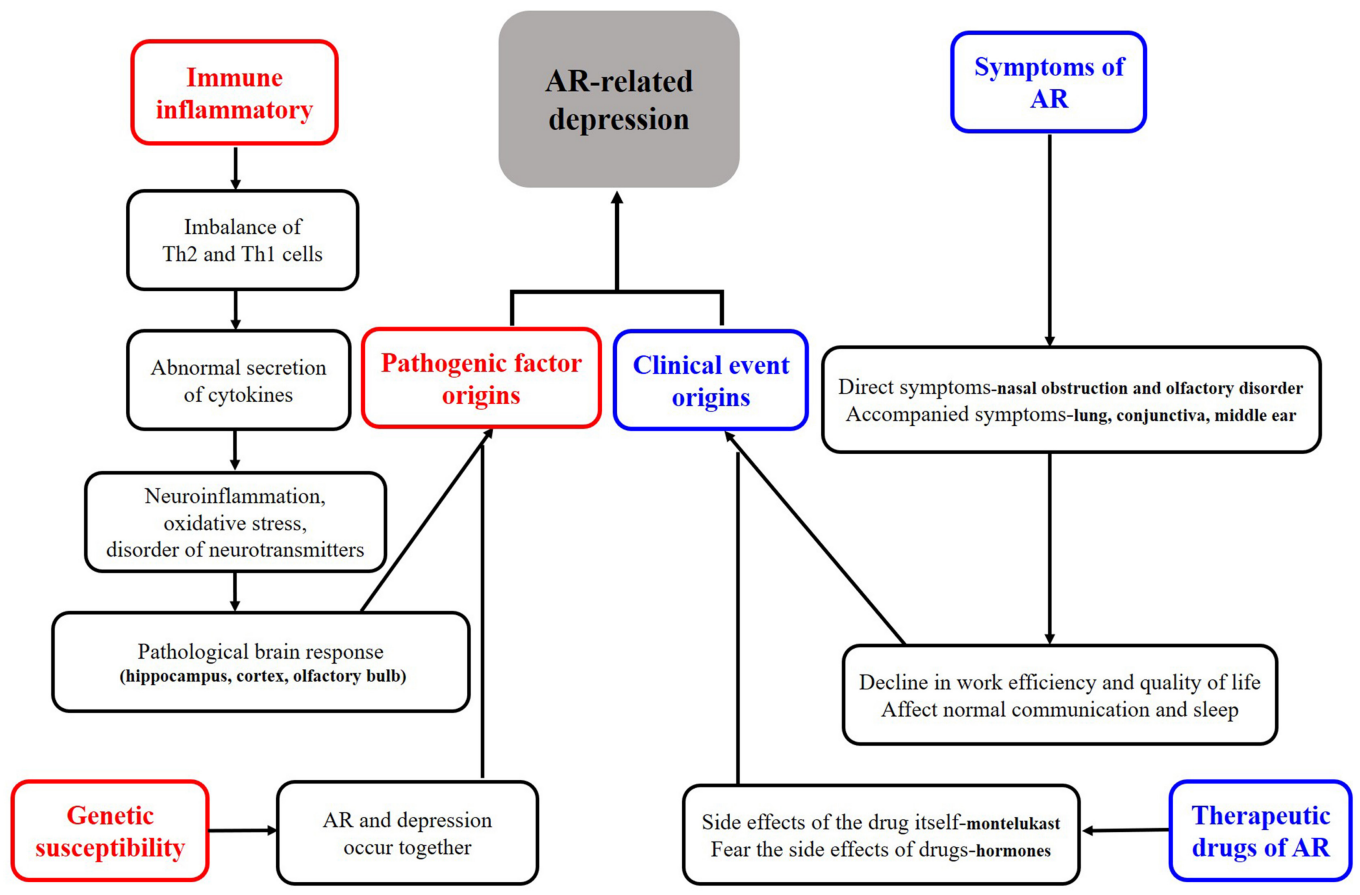
Classification of depression in AR based on possible pathogenesis.

As shown in Figure 1, we tried to define the roles of depression in AR. Firstly, all the clinical cases of depression in AR can be collectively referred to as “depression related to AR.” More specifically, the definition of depression can be divided into three categories, (a) when depression is considered “as a risk factor,” it can be called “AR associated with depression.” There is no report that depression directly causes AR. Although many studies have suggested an increased risk of asthma and allergic diseases (e.g., AR) in children born to mothers with a prenatal history of psychosocial stress (e.g., depression) (8–11), whether depression can directly cause AR deserves further investigations. (b) When depression is considered as “a secondary symptom or disease,” it can be defined as “depression secondary to AR,” as demonstrated in many studies (6, 7, 12–14). It is also reported that the development of depressive symptoms increases the severity of AR and the rate of treatment failure and significantly raises social expenditure on AR. Therefore, this condition is most worthy of further investigations. (c) It may be defined as “AR with depression” when it is considered as a co-morbidity. Theoretically this condition does exist, and the possibility of co-morbidity of AR and depression has been suggested in studies on genetic susceptibility in twins (3, 15, 16). However, none of the studies retrieved has independently reported this condition, and we assume that many studies might have classified it into the second category. Therefore, a clear definition of this condition is essential for future studies. Secondly, whatever condition mentioned above is unfortunate for an individual patient. Clinically, the second category, i.e., “depression secondary to AR,” is most urgent to be addressed. Based on the baseline number of 500 million AR patients globally and the minimum incidence of depression of 20% among these patients, literally at least 100 million patients with depression secondary to AR need help. Thirdly, no guidelines on the depression in AR have been available in any country, except that montelukast may trigger depression as a very rare side effect, which was most frequently mentioned (17, 18). Thus, we define and classify the roles of depression in AR, which may enhance the awareness of depression in AR among medical staff and promote the development of guidelines for diversified treatment protocols, thus achieving the precise treatment of depressed AR patients. Unfortunately, few studies have elucidated the classification of the roles of depression in AR, especially the “depression secondary to AR.” Nevertheless, by summarizing its causes and mechanisms, we classified the depression in AR into pathogenic factor origins and clinical event origins, as shown in Figure 2. Of course, here we kindly remind everyone that the depression we mention in this article refers to major depression (19).

## Causes and Mechanisms of Depression in AR

As shown in Figure 2, causes of depression in AR are described mainly in four aspects, namely related to symptoms of AR, related to treatments of AR, related to genetic influence, and related to immune-inflammatory. It has been well-recognized that AR symptoms are the most obvious and direct cause of depression in AR. The severity, duration, and comorbidities of AR will affect the mental status and lead to irritability, low mood, sadness, and finally depression. It was found that the risk of depression was 2.7-fold higher in patients with severe AR than in those with mild AR (20). Whether the risk of depression differs between patients with perennial AR and those with seasonal AR remains controversial in terms of symptom persistence (21), and the potential difference may be explained by the different indicators and sample size in different studies. More specifically in manifestation of symptoms it was found that nasal congestion, nasal itching, and olfactory disturbances contributed significantly to the development of depression in AR patients (22–24), which may be related to the fact that such symptoms, especially nasal congestion, can have a serious impact on sleep (23). In fact, when AR is accompanied by other diseases such as sinusitis (25) and asthma (26), the patients will have a higher depression scale score than AR patients without comorbidities; in addition, these patients are prone to suicidal behavior.

Two major factors are involved in the depression caused by AR treatment: (a) excessive concern about the side effects of the treatments; in particular, about 66% of AR patients developed depressive symptoms after long-term use of glucocorticoids (27); and (b) depression caused by the medication itself. Although the risk of depression associated with montelukast use has been mentioned in several guidelines (17, 18), the incidence of depression in such AR patients using montelukast is roughly negligible. Nevertheless, assessment before medication and monitoring during drug administration are essential in some special and sensitive populations.

In terms of genetic susceptibility, some Chinese authors (3) suggested that genetic factors, especially maternal inheritance, may play a key role in the association between AR and depression, and agreed on the possibility that the ADCYAP1R1 gene (28) deserves attention; however, studies on a single gene are limited, and the potential role of epigenetic research warrants further investigations.

Finally and most interestingly, immune-inflammatory response may also play a key role. As is known, AR essentially is an IgE-mediated immune-inflammatory response triggered by allergens. Many studies have suggested that peripheral inflammatory signals or cytokines (e.g., IL-1β, IL-6, TNF-α, GM-CSF, and IL-5, IL-13, and IL-4) in the nose can enter the central system through the possible “neural pathways (olfactory and trigeminal nerves),” “cellular pathways,” and “humoral pathways,” causing neuroinflammation, oxidative stress, and neurotransmitter disturbances in the brain, ultimately leading to depression in individuals with AR (29–31). Notably, most of the above findings were based on animal models, as there are ethical regulations governing the conduct of research involving human subjects with AR-related depression, especially when invasive manipulations are involved. Fortunately, as a non-invasive examination tool, functional magnetic resonance imaging (fMRI) has been applied to study the brain response in AR patients (32, 33), which provides the possibility to further directly study brain alterations in depressed AR patients. In recent years, China maintains a leading position in animal studies on the mechanisms of depression in AR. As early as 2014, Chinese scientists (30, 31) explored the effects of environmental pollutants on allergic inflammation-mediated depression using mouse models. They found that when mice were exposed to the environmental pollutant dibutyl phthalate, the risk of developing depression was higher in allergic mice and was thought to be related to oxidative stress. Subsequently, in 2016, other researchers in China (29) found that some depressive-like behaviors were observed in AR mice during continuous allergen exposure and that the appearance of these depression-like behaviors was associated with the inhibition of lateral septum dorsal part (LSD) and paraventricular nucleus (PVN) neurons and the activation of microglia in the cingulate cortex; however, such depression-like behaviors returned to normal after long-term allergen avoidance. In 2018, some authors in other countries (34) reported that the depression-like behaviors in ovalbumin (OVA)-sensitized juvenile rats were related to hippocampal oxidative stress and that exercise improved the antioxidant system and thus alleviated these behaviors. There have been no other animal studies on AR-related depression in recent years. However, some animal studies on abnormal brain responses secondary to nasal immune-inflammatory responses after AR have been reported, and it has been suggested that brain regions such as hippocampus, cortex, and olfactory bulb are closely related to the development of mental disorders in AR animals (35–37). Along with the achievements in animal model studies, some authors have also conducted relevant experiments using blood samples and cells from depressed patients and AR patients, and confirmed the correlation between the neurotransmitter 5-hydroxytryptamine and the cytokines secreted by T lymphocytes during an allergic reaction (38).

## Diagnosis and Treatment of Depression in AR

The diagnosis of depression is often missed, even in specialist institutions and clinics (39). There is no doubt that the recognition, diagnosis, and treatment of depression are more challenging for rhinologists and allergologists. Therefore, identifying the co-existing depressive symptoms in AR patients is a priority. In the absence of relevant guidelines, the diagnosis of depression in AR should be based on the typical clinical manifestations (e.g., low mood) of depression in AR patients, along with the use of standardized scales for clinical screening and assessment of depression. The diagnosis of depression can be made according to the criteria in the International Classification of Diseases (ICD)-10, which consists of three core symptoms and seven associated symptoms, and the disease can be further classified into mild, moderate, and severe, depending on the severity of the symptoms (40). Evaluation of clinical symptoms and self-rating scales are valid and convenient tools in screening depressed AR patients (3). Currently, most of the self-rating scales can be filled out within 10 min. However, it is important to remember that self-reported depression is indicative of, but not confirmation of a depressive episode that meets diagnostic criteria. In other words, rating scales of depression could identify “probable cases of depression,” but not “depressive disorders.” Symptomatic constellation of self-reported complaints is not a diagnosis of “depression” due to bias is unavoidable to emerge with self-rating scales. So, for patients with more complex conditions, examiner-rating scale may be used, especially patients with severe symptoms, in addition to providing treatment strategies for AR, rhinologists and allergologists should recommend strongly that the patient should seek help from a psychiatrist in a specialized institution. After all, psychiatric examination, that is, structured clinical interview by a trained clinician, has the last word in the diagnosis of depression, not the scale.

Although there is a lack of guidelines and consensus on the treatment of depression in AR, some clinical studies have found that untreated depression exacerbates the symptoms of AR, suggesting that the proper management of depression is important for the comprehensive treatment of AR patients (41). Clinical studies have also shown that interventions for depression in AR may include medications, psychological interventions, vitamins, probiotics, and immunotherapy, all of which can reduce the depression scores and improve the quality of life (3). What should be stressed is that, pathogenic factor origins and clinical event origins should be managed differently, in some cases, effective results can be achieved after merely controlling the nasal symptoms of AR; however, in some other cases, multiple interventions should be given, although they may fail. In addition, research has shown that antidepressants such as escitalopram (42) and desipramine (43) can restore T-cell imbalance and alleviate inflammatory response in AR patients; thus, whether these drugs can play a dual role in treating AR and depression warrants further research.

## Conclusions

The number of AR patients is increasing as we are being exposed to increasingly polluted environments (44). Meanwhile, the incidences of various mental disorders (especially depression) continue to rise with the increasing stress at home and workplace (45). The incidence of depression in AR is between 20% and 40%. Based on the number of about 500 million AR patients worldwide, the number of patients with depression in AR is estimated to be 100–200 million, so the demand for treatment of depression in AR is huge, especially depression secondary to AR. Although depression in AR has been noted and some treatment options have been attempted, there are many ethnic and legal problems concerning the invasive investigations in human subjects; in addition, preclinical studies in animal models are limited. Therefore, there are still many issues to be addressed, especially the key brain regions, cells, and molecules that respond early after the depression occurs. In order to avoid the tragedy of “allergy in adolescence and use of antidepressants in middle age” (46), more basic and clinical research on depression in AR should be conducted. Specifically, in animal research, it can try OVA + CUMS (chronic unpredictable mild stress) to build a compound model of AR + depression to further explore its cytological and molecular mechanisms. In clinical research, since self-reported rating scales is not a trust worthy methods of evaluation, an improved methodology to assess depression among patients with AR is necessary. And, more to the point, if more and more case-control studies or even cohort studies can be carried out, it will be conducive to the development of relevant guidelines and consensuses.

## Author Contributions

X-CS: conception and design. CR: administrative support. YZ, W-BZ, and H-RW: provision of study materials. W-BZ, H-RW, and Y-KM: collection and assembly of data. CR and X-CS: data analysis and interpretation. All authors: manuscript writing and final approval of manuscript.

## Funding

This study was supported by National Natural Science Foundation of China (Grant No. 82071021), the Key Research and Development Program of Shandong Province (Major Science and Technology Innovation Project) (Grant No. 2020CXGC011302), the Key Project of Shandong Provincial Natural Science Foundation (Grant No. ZR2020KH024), and the Natural Science Foundation of Shandong Province, China (Grant No. ZR2020MH177).

## Conflict of Interest

The authors declare that the research was conducted in the absence of any commercial or financial relationships that could be construed as a potential conflict of interest.

## Publisher's Note

All claims expressed in this article are solely those of the authors and do not necessarily represent those of their affiliated organizations, or those of the publisher, the editors and the reviewers. Any product that may be evaluated in this article, or claim that may be made by its manufacturer, is not guaranteed or endorsed by the publisher.
